# A geometric network model of intrinsic grey-matter connectivity of the human brain

**DOI:** 10.1038/srep15397

**Published:** 2015-10-27

**Authors:** Yi-Ping Lo, Reuben O’Dea, Jonathan J. Crofts, Cheol E. Han, Marcus Kaiser

**Affiliations:** 1 School of Science and Technology, Nottingham Trent University, Nottingham, NG11 8NS, UK; 2 Centre for Mathematical Medicine and Biology, School of Mathematical Sciences, University of Nottingham, Nottingham, NG7 2RD, UK; 3 Department of Mathematics, National Taiwan University, Taipei, Taiwan 10617; 4 Department of Brain and Cognitive Sciences, Seoul National University, South Korea; 5 Interdisciplinary Computing and Complex Biosystems (ICOS) Research Group, School of Computing Science, Newcastle University, Newcastle upon Tyne, NE1 7RU, UK; 6 Institute of Neuroscience, Newcastle University, Newcastle upon Tyne, NE2 4HH, UK

## Abstract

Network science provides a general framework for analysing the large-scale brain networks that naturally arise from modern neuroimaging studies, and a key goal in theoretical neuroscience is to understand the extent to which these neural architectures influence the dynamical processes they sustain. To date, brain network modelling has largely been conducted at the macroscale level (*i.e.* white-matter tracts), despite growing evidence of the role that local grey matter architecture plays in a variety of brain disorders. Here, we present a new model of intrinsic grey matter connectivity of the human connectome. Importantly, the new model incorporates detailed information on cortical geometry to construct ‘shortcuts’ through the thickness of the cortex, thus enabling spatially distant brain regions, as measured along the cortical surface, to communicate. Our study indicates that structures based on human brain surface information differ significantly, both in terms of their topological network characteristics and activity propagation properties, when compared against a variety of alternative geometries and generative algorithms. In particular, this might help explain histological patterns of grey matter connectivity, highlighting that observed connection distances may have arisen to maximise information processing ability, and that such gains are consistent with (and enhanced by) the presence of short-cut connections.

The study of real-world systems naturally leads to large-scale complex networks that support a variety of emergent dynamical phenomena. Yet, despite the plethora of network models describing the topology and dynamics of such systems^1^,^2^, the precise relationship between observed structural and functional connectivities remains an important open problem in network science. In neuroscience, in particular, a current challenge is to better incorporate physiological information, thus obtaining improved structural network models capable of supporting dynamics that more accurately reflect observed neural activity^3^,^4^.

Consisting of some 10^10^ neurons and 10^14^ connections all embedded within a highly constrained anatomical space^5^, the human brain is perhaps nature’s most complex system. Such constraints have a considerable impact on the organisation of both brain anatomy and connectivity and, alongside a number of important biological and physical factors, naturally give rise to the cerebral convolutions observed in the human cortex. Local cortico-cortical connectivity, in particular, has been proposed as a possible explanation for the folded, three-dimensional structure of the cerebral cortex, with axonal tensions between densely/sparsely connected sets of neurons hypothesised to form gyri/sulci^6^,^7^. Such connections are well-documented in histological studies, and may extend for distances of 4–5 millimetres^8^,^9^,^10^. It is perhaps surprising then, that the majority of network connectivity studies to date have concentrated on long-range connectivities obtained via modern neuroimaging techniques capable of inferring white matter paths^11^,^12^,^13^, whilst grey matter architecture (that is, local network structure confined to the cortical sheet), has largely been ignored. While computational models often concern the micro-scale within cortical columns^14^,^15^ and the macro-scale of fibre tract connectivity^16^, the meso-scale of connectivity^17^ between columns along the cortical surface has received little attention.

Recent studies suggest that myriad neurological conditions (epilepsy, schizophrenia and Alzhiemer’s to name a few) are accompanied by abnormalities in both gross anatomy and structural connectivity of the brain^13^. In particular, there is emerging evidence that alterations in cortical folding are present within a variety of brain disorders^18^,^19^,^20^, and thus investigations into the relation between surface morphology and brain connectivity could provide novel insights into these disorders.

To address this deficiency in the literature, a small number of recent studies have begun to employ cortical geometry as a proxy measure for grey matter architecture, in order to infer large-scale network connectivity^21^,^22^,^23^. Importantly, these investigations have evidenced a number of important connections between network topology (inferred via network features of relevance to spatially constrained systems, *e.g.*, clustering coefficient, characteristic path length and modularity) and cortical folding, and as such, promise to shed further light on the relationship between neuroanatomy and brain dynamics. For example, O’Dea *et al.*^22^ found that key features of relevance to seizure initiation and progression (in particular, those highlighting the importance of the site of initiation within the network) are poorly captured by standard, planar network models when compared against a spatially embedded network representing the convoluted structure of the cortical surface. Further evidence of the important role that cortical geometry plays in shaping neural connectivity is provided by Ecker *et al.*^24^. Here the authors used surface geometry to model local cortico—cortical structure, and in so doing, were able to show that observed abnormalities in brain connectivity of patients with autism spectrum disorder were accompanied by altered neural connections in both grey and white matter. We note here that in the present work we are concerned with networks whose nodes are to be interpreted as ‘neural units’ comprising many neurons, and representing a single cortical column, say. Detailed consideration of synaptic signalling models or neural connectivity footprints (see *e.g.* Coombes^25^, Voges and Perrinet^26^ and references therein) is therefore not appropriate.

Whilst the influence of network structure on network dynamics is well documented^2^,^27^, studies such as those highlighted above demonstrate the importance of understanding the extent to which geometry influences not only cortical network construction, but also the ongoing and evoked neural dynamics these brain structures underlie.

We employ MRI data from a cohort of human subjects obtained from the NKI dataset^28^ to define a neural network, representing grey matter connectivity and whose structure is related to the folded structure of the cortical surface through the existence of shortcut connections through the thickness of the cortical sheet. We effect this via a simple connectivity rule based on Euclidean distance, but modified to prohibit connections that correspond to excessively long geodesic paths or, equivalently, those which correspond to long-range connections between adjacent gyri/sulci through the white matter (so-called U-fibres, which may extend for distances of 1–3 cm^29^): the latter being crucial to the construction of a network whose connectivity footprint is of physiological relevance to grey matter cortical structures. We remark that this work is therefore distinct both from that presented in Henderson and Robinson^23^, which considers the link between cortical architecture and white matter connectivity, and Ecker *et al.*^24^, in which only tangential connections are considered. Of course, a complete description of cortical network dynamics would require both long-range (white matter) connectivity and grey matter architecture to be included; however, to reemphasise, we concentrate here only on the dense and short-range connections that exist within the cortical sheet and investigate aspects of these mesoscale networks in detail. The focus therefore differs from the majority of network connectivity studies which concentrate on long-range, white matter connectivities obtained from modern neuroimaging techniques. To study the activation of such a network, we employ a simple spreading model of the type studied in O’Dea *et al.*^22^, Kaiser *et al.*^30^ and Mišić *et al.*^31^ in favour of the more complex descriptions employed in the theoretical neuroscience literature. Such a deliberately abstract approach emphasises the emergence of global patterns, thereby allowing clear investigation of the influence of the network architecture in isolation from the dynamics that it sustains. Despite not being biophysical in nature, this threshold-based automaton model may be viewed as a coarse description of neural activation. The activation process may be thought of as the spreading of synchrony: if many neighbours of a neural mass enter a particular oscillatory regime, they act to promote synchrony on that mass, leading to an oscillatory cascade^31^.

To highlight the impact of our network construction method and the underlying cortical geometry on the resulting network structure, we employ our new method to construct cortical networks for a large cohort of 121 healthy brains (obtained from the NKI dataset^28^). Additionally, we study in detail the network that arises from a sequence of increasingly smooth cortical surfaces (obtained by deforming, or ‘inflating’ the cortical surface so that it becomes sequentially less folded, while conserving area), and compare networks arising from our method against similar structures constructed according to Euclidean or geodesic distance only—the former not adhering to physiological connectivity constraints in the grey matter, the latter, not considering shortcuts.

Our analyses indicate that the inclusion of shortcuts associated with cortical folds impacts significantly on the structure of the resulting network. Such networks display significantly higher clustering and reduced characteristic path length, indicative of increased efficiencies in information processing capacity. We remark that similar increases are observed in networks arising both from our method, and those constructed according to Euclidean distance only; however, our method restricts to a more physiologically-relevant connectivity footprint. Importantly, we observe that maximal clustering and significant reduction in characteristic path length (by 75%) occurs in the connectivity range (approx 4 mm) observed in histological studies^8^,^9^,^10^. Our networks therefore offer evidence that cortical connectivity structures have arisen such that their network topological properties maximise communication efficiency while minimising associated wiring costs, and therefore can be considered as archetypal examples of well-studied spatial networks^32^.

Under simple assumptions on the activation dynamics of such networks, we show that these shortcuts lead to differences in the propagation of activation through the network. Furthermore, we observe that a dependence of network activation speed on initial activation site is imbued by the detailed network structure.

The results of our work therefore demonstrate that dynamics of relevance to epileptic seizure initiation and propagation are significantly influenced by the cortical network structure that we choose, and such considerations should therefore be included in theoretical models that aim to provide a more complete description of neural network dynamics, and epileptic seizure activity, in particular.

The remainder of this article is organised as follows. In the Methods section we employ MRI data to construct a network architecture embedded in the surface of the human cortex, accounting for cortico—cortical shortcuts and define a simple cellular automaton-like rule governing the activity in such a network. In Results, we perform numerical studies to highlight how cortical folding, and the inclusion of shortcuts, influences network structure and activation, when compared to smoother structures and simpler network construction algorithms. Additionally, the variability of these results in a large cohort of human subjects is indicated. We provide a summary of our results in the **Discussion**, together with a discussion of their relevance, and highlight possible future developments.

## Methods

In this paper, we seek to investigate the influence of the gyrification of the human cortex on both network structure and activation spreading dynamics over the cortical surface. To achieve this, we employ MRI data to define a network, whose nodes may be thought of as analogous to cortical columns, and whose edges represent the short-range lateral connections which are prevalent in the grey matter of the human cortex. We study the propagation of activation through this network, governed by a simple spreading model^22^,^30^, described below.

The model that we describe below embeds the idea that due to the highly folded nature of the cortical surface, cortical regions that are distant as measured on the cortical surface may be connected by cortico—cortical ‘shortcuts’ through the thickness of the cortical sheet. Our network is therefore distinct from those analysed in (*e.g.*) Henderson and Robinson^23^, Ecker *et al.*^24^ which consider white matter, or purely tangential connections, respectively.

### Network construction

We employ MRI data resulting from a study by the Nathan Kline Institute (NKI)^28^ which is freely available via the following public online database: http://fcon_1000.projects.nitrc.org/indi/pro/nki.html. In total we included 121 participants aged between 4 and 40 years. Cortical reconstruction was performed using the Freesurfer image analysis suite, which is documented and freely available online (http://surfer.nmr.mgh.harvard.edu/); the algorithms employed for this construction are discussed elsewhere^33^,^34^. In brief: a single filled volume was generated for each hemisphere onto which a triangular surface tessellation is fitted, resulting in a mesh or lattice of spatial coordinates defining the grey matter surface (also termed pial surface) of approximately 150,000 vertices (the smallest lattice obtained from the NKI dataset comprises 115,390 vertices, the largest, 187,126). No manual edits were necessary.

We employ the triangulated lattice described above as a basis from which to define the cortical networks used in the remainder of the study.

**Figure 1:**
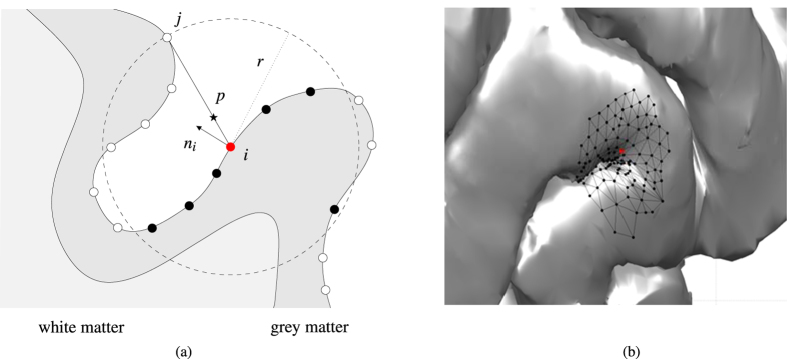
Cortical network construction. (**a**) Unphysical connections, which are near in ambient space but excessively distant as measured on the cortical surface, are removed by exploiting the signed distance method^35^ to highlight connections which correspond to crossing ambient space within the connection distance *r*. The exterior point *p* lying between the central node *i* (highlighted in red) and the target node *j* is marked with a star. Target nodes that remain connected to node *i* under this rule are shown as filled black circles. (**b**) A sample of the triangulated lattice defining the cortical surface, indicating the connectivity of a node (highlighted in red) in a network constructed via the process described in **Network construction**.

Our network construction approach is designed in order to allow ‘short-cuts’, induced by the folded architecture, to exist; *i.e.* we do not restrict attention to purely tangential connections, measured on the cortical surface (as in Ecker *et al.* [24]) However, due to physical ‘wiring cost’ considerations, connections which correspond to excessively long geodesic paths must be prohibited. To effect this, we add additional links to the minimally-connected nearest-neighbour lattice according to the following rationale: (i) vertex pairs are connected if they lie within a specified Euclidean distance, *r* of each other; and (ii) ‘unphysical’ connections, which are near in ambient space but excessively distant as measured on the cortical surface, are removed. See Fig. 1. The highly convoluted structure of the cortical surface means that the latter condition is essential to the construction of networks suitable for the representation of short-range cortico–cortical connections. A convenient method with which to effect this is to remove connections which correspond to Euclidean connections crossing ambient space: a specific Euclidean distance *r* effectively places a scale on the allowable wiring cost for a given connection; as illustrated in Fig. 1, links between vertices which cross ambient space (as distinct from short-cuts which lie within the cortical sheet) imply very long connection distances and should be rejected. We remark in passing that the choice of *r* is key to the physiological relevance of the resulting network—the dense connectivity footprint associated with each node in our network is appropriate for local cortical connections (including both the tangential connections and shortcuts occuring within the cortical sheet); however, for sufficiently large *r*, such a network will include connections between more distant cortical regions, which employ (*e.g.*) U-fibres extending into the white matter (*e.g.* those analysed in Henderson and Robinson^23^ and elsewhere), and whose connectivity footprint may not adhere to our assumptions. In the numerical experiments that follow, we focus our attention on values of *r* in line with physiologically-relevant connection distances discussed in the previous section.

An efficient way to detect spurious connections according to the above consideration is to employ the signed distance method^35^, exploiting the angle-weighted pseudo-normal to the cortical surface. This method is integral to the proper construction of our cortical network, and so below we summarise the algorithm in some detail (extensive treatment of the theoretical considerations is given in Baerentzen and Aanaes^35^, and citations therein).

1. We consider a vertex *i* (with position vector *x*_*i*_) and a set of *N* target vertices *x*_*j*_ 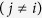, connected according to rule (i) above (and hence lying within a sphere of radius *r* centred on *x*_*i*_).

2. We isolate the *m* vertices 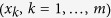 associated with cortical folds leaving and subsequently re-entering the Euclidean connection region. More precisely, the set {*x*_*k*_} consists of the subset of target vertices *x*_*j*_ for which the shortest path (calculated using the nearest-neighbour mesh defined above) from vertex *j* to *i* visits at least one vertex a distance greater than *r* from *x*_*i*_.

3. For each target vertex 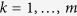, we consider the point


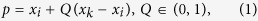


lying between *i* and *k*. We use *Q* = 0.1 in the networks that we construct in the remainder of this paper; our numerical investigations indicate that such a choice ensures that the point of interest, *p*, is sufficiently near to the mesh surface that it is not unduly influenced by folds lying between *x*_*i*_ and *x*_*k*_.

4. We compute the *signed distance* for each of these target vertices:


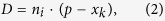


wherein *n*_*i*_ is a suitable normal to the surface at node *i* (details below). The sign of *D* dictates the position of *p* relative to a 3D mesh surface via the following conditions:


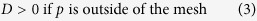



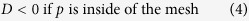



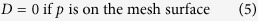


If the signed distance *D* associated with a target vertex *k* is negative, *p* is outside of the mesh and we conclude that connecting the vertex *i* to *k* involves crossing ambient space; see Fig. 1. As discussed in the introduction, such connections represent (equivalently) an excessively long geodesic connection, relative to *r*, or a connection which connects adjacent gyri/sulci via a U-fibre entering the white matter. As we restrict attention to connections within the cortical sheet here, we therefore reject such a connection.

Key to computing the signed distance *D* in the above method, is the normal to the cortical surface. We employ the angle-weighted pseudonormal^36^,^37^ which captures appropriate normal properties, even at the edge and node gradient discontinuities present in a triangular mesh, and defined as follows. Considering a mesh vertex *i*, with *F* incident faces (with unit normals *n*_*m*_, 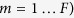, the angle-weighted pseudonormal at the vertex is:


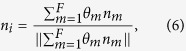


where *θ*_*m*_ is the angle of the *m*^*th*^ mesh triangle incident to the vertex *i*. This definition generalises naturally to faces (*F* = 1, *θ*_*m*_ = 2*π*) and edges (*F* = 2, *θ*_*m*_ = *π*), and in contrast to other commonly-employed pseudonormals, may be used to determine uniquely whether a point lies within such a polyhedral object (see Baerentzen and Aanaes^35^, and citations therein).

### Network characterisation

Systematic comparison of the cortical networks considered here is effected via the following standard measures, common in the network science literature: (i) the average degree; (ii) the clustering coefficient, which is given mathematically as


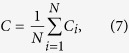


where *C*_*i*_ denotes the probability that any two neighbours of node *i* are connected and *N* the number of nodes in a graph; and (iii) the characteristic path length, *L*, which is defined as the number of edges in the shortest path between two vertices, averaged over all pairs of vertices.

In addition, to characterise the level of cortical folding (or gyrification) of a brain, we employ the Gyrification Index (GI). This whole-brain measure expresses the relative size of the cortical surface, to that which is superficially exposed. For the brains under consideration, this is calculated as the ratio of the pial surface to that of the convex hull, enclosing the cortex. We remark that this is a simple 3D extension of the classical GI calculation (see Zilles *et al.*^38^), which employs 2D coronal slices. Precise spatial detail is obtained via the local Gyrification Index (lGI), which provides a GI value for each vertex in the cortical mesh, by calculating the GI associated with a region of interest surrounding each vertex. In brief, the lGI of a vertex is computed as the ratio of the surface area of the folded, or buried, cortical surface to the outer, exposed surface included within a sphere of radius 25 mm, centred at the vertex of interest^39^. These measures are calculated via the FreeSurfer software package.

### Network activation

In addition to network analyses, we study the influence of the network structure on node activation dynamics via a simple spreading model^22^,^30^ summarized as follows.

Nodes *i* are restricted to exist in one of two states: active (*x*_*i*_ = 1), or inactive (*x*_*i*_ = 0). Starting from an initial activation state, simulation operated in discrete timesteps; from one timestep to the next, an inactive node became activated (or an active node remained in the active state) if it was connected to at least *m* active nodes. Initial conditions comprised a small region of activation (1% of the total nodes in the network) surrounding a node selected at random. We choose the mean fraction of activated nodes as our key metric with which to investigate the different networks; ensemble measures of network dynamics were constructed from 20,000 realisations.

## Results

We conduct two separate numerical investigations:

### 1. Cortical inflation

To highlight in detail the influence of cortical folding on network structure, and activation dynamics, we choose a brain at random from the NKI dataset and deform it so that it becomes sequentially less folded. These ‘inflations’ are performed via the FreeSurfer software package and are performed such that the total area of the inflated cortical surface is conserved. For each inflation, we study in detail both the network structure arising from our method and the corresponding network activation properties, as described in **Network characterisation** and **Network activation**.

### 2. Cohort variability study

To investigate inter-subject variability in our cortical networks due to differences in brain structure, we compare the activation of networks constructed from all brains in the NKI cohort.

In all of the above numerical experiments, a range of values of *r* is chosen to highlight the variability of network structure and activation dynamics; however, our concentration is on values lying within a physiologically-relevant regime (as noted in the introduction, the cortico—cortical connections that we consider can extend for distances of 4–5 mm). The activation parameter is fixed as *m* = 2 without loss of generality. This value places a lower bound on the connectivity of the network for which network activation can occur, and influences the speed of spreading of activation in the network (and, together with the value of *r*, the shape of the advancing activation front). Since we consider highly simplified dynamics in this study, omitting, for example, random inactivation or complex intra-node dynamics (the better to emphasise the importance of network structure on activation dynamics), the balance between *m* and *r* determines completely the speed of activation of the network (indeed, for appropriate *m* and *r*, whole network excitation is inevitable) and, furthermore, affects all networks in the same manner.

## Network statistics

**Figure 2:**
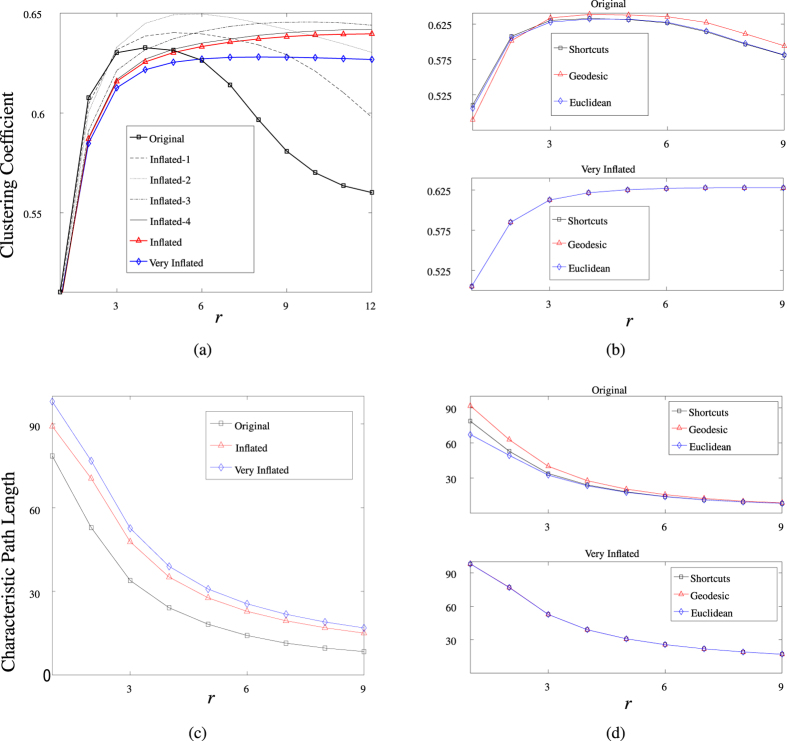
Network measures. (**a**) The clustering coefficient and (**c**) the characteristic path length plotted as a function of the connectivity distance *r* for the original, inflated and very inflated brains. The additional lines in (**a**) display the results for intermediary inflations obtained from the deformation algorithm as it steps sequentially between the original and inflated brains. (**b**) A comparison of the clustering coefficient and (**d**) the characteristic path length for networks constructed according to our new, shortcut-based algorithm introduced in this work, and those constructed according to Euclidean and geodesic distances only.

Figure 2 shows the network measures *C* and *L*, with which we quantify the effect of cortical folding on local network architecture, as a function of connectivity distance, *r*.

**Figure 3:**
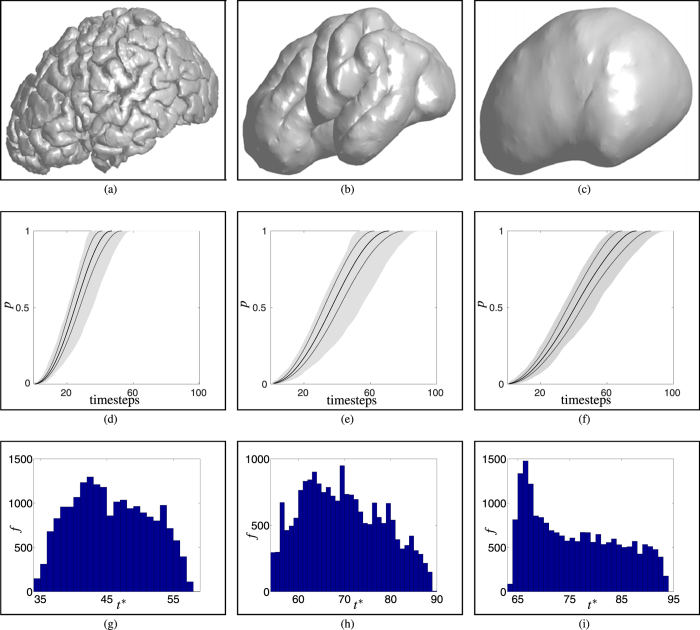
Cortical inflation study. (**a**) A cortical surface taken from the NKI dataset^28^; (**b**,**c**) inflated versions of this structure: an ‘intermediate’, with folding still evident, and a ‘very inflated’ structure with no folds. (**d**–**f**) The evolution of the mean fraction of activated nodes 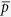 (solid lines), together with a confidence interval of width 2*σ* (dotted lines), and the spread of activation fractions *p* in each realisation (shaded area). (**g**–**i**) Histograms showing the distribution of the times to full network activation, t*, obtained from 20,000 simulations in each network.

We compare these topological characteristics for a range of cortical structures, concentrating on an undeformed cortical surface (Fig. 3(a)), a partially-inflated structure, with cortical folds still evident (Fig. 3(b)) and a very inflated, smooth surface (Fig. 3(c)); in Fig. 2(a) we also include intermediate inflations for completeness. For each of the above structures a connectivity, or adjacency, matrix 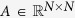 was constructed according to the shortcut algorithm described in **Network construction**. In the example considered here *N* = 145, 434. Considering Fig. 2(a,c), we observe the following: (i) For increasing values of *r* the inclusion of long-range connections between adjacent gyri (akin to U-fibres) results in a non-monotonic clustering profile: for small values of *r* the dense connectivity footprint typical of nodes lying within the gyri ensures a relatively high level of clustering, as the likelihood that neighbour nodes are also connected is high; however, as *r* increases, this likelihood diminishes due to the increased chance that neighbour nodes lie in adjacent gyri. The clustering coefficient profiles for the intermediate inflations included in Fig. 2(a) further highlight the effect of cortical geometry on the clustering profile. (ii) Whilst the effects of connectivity distance on characteristic path length are qualitatively similar across all three network structures (Fig. 2(c)), the additional links induced by cortical folds in the original undeformed surface, and to a lesser extent the inflated cortical surface, lead to significantly larger reductions in network size, as measured by path length based measures (*e.g.* characteristic path length). It is worth noting at this point, that due to the exclusion of long-range projection fibres from our analysis, the observed path lengths in Figure 2 are much higher than in previous studies reporting small-world-like features.

**Figure 4:**
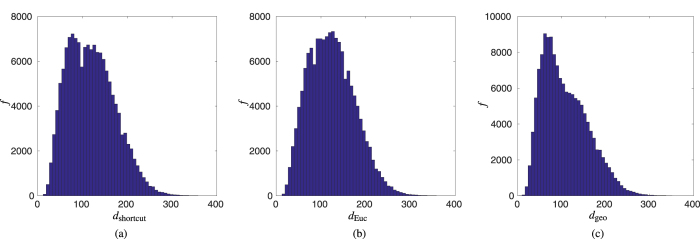
Degree distributions. Comparison of the degree distributions for the networks constructed according to (**a**) our new, shortcut-based algorithm; (**b**) Euclidean distance; and (**c**) Geodesic distance.

In addition to the inflation study conducted above, we compared the clustering coefficient and characteristic path length for networks constructed using our new, shortcut based algorithm, and those resulting from consideration of Euclidean and geodesic distances only (Fig. 2(b,d)). These results indicate that for the original undeformed brain the resulting networks exhibit distinctly different features, whilst for inflated (and intermediate—data not shown) structures the resulting networks are comparable. As expected, characteristic path length is significantly reduced in those networks for which Euclidean shortcuts are permitted in the construction process as opposed to those built using the more stringent, geodesic constraints. For example, for *r* = 4 mm, we find that the characteristic path length of a typical geodesic network is approximately 15–20% greater than it is in the corresponding Euclidean or shortcut based network structure. Differences in network structure are further manifested by the degree distributions (Fig. 4(a–c)), with average degree for the shortcut, Euclidean and geodesic networks, again with *r* = 4, given by 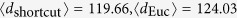 and 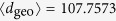, respectively. Additionally, we note that these distributions are all right skewed with respective skewness values of 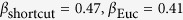 and 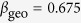, with the geodesic network, in particular, displaying a long tail towards high degrees, due to the existence of fewer highly connected nodes. (Sample skewness values were computed using the Matlab Statistics Toolbox).

These network analyses indicate that the inclusion of communication shortcuts associated with cortical gyrification results in a network with significantly higher clustering and reduced characteristic path length, properties indicative of increased efficiencies in information/signal processing capabilities. Moreover, in networks corresponding to the undeformed cortex, we observe maximal clustering at 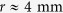 at which point the characteristic path length is reduced by approximately 75%. As noted in the introduction, cortical connections observed in histological studies may extend for 4–5 mm; this correspondence suggests that such a connectivity distance may have arisen to provide an optimal balance between communication efficiency and the associated wiring costs. Furthermore, the presence of our hypothesised shortcut connections leads to significant gains in information processing capacity. We remark that such gains are also seen in networks constructed according to Euclidean distance only; however, such a network is unlikely to display physiologically-relevant connectivity footprints due to its inclusion of connections more appropriately described by white matter connection models.

In summary, we suggest that our networks provide an indication that grey matter connectivity structures could have arisen in order to maximise information processing ability, and that such gains are consistent with (and enhanced by) the presence of short-cut connections.

## Network Activation Dynamics

### Cortical structure

Figure 3 summarises the results of our cortical inflation study, indicating how the structure of the network (described in detail in **Network statistics**), and the initial point of activation, influences the spread of activation (measured by the fraction of activated nodes in the network, denoted *p*) in the three example networks considered in the previous section, *i.e.* an undeformed cortical surface, a partially-inflated structure, and a very inflated, smooth surface (see Fig. 3(a–c)). The simulation results shown in Fig. 3(d–f), and the remainder of this paper, correspond to *r* = 4 (for discussion of suitable choice of connectivity distance, see **Introduction** and **Network statistics**).

Figure 3(d–f) show the time evolution of the mean fraction of activated nodes in each network. Also shown is a confidence interval of width 2*σ* and the spread of the individual trajectories, together indicating the variability in activation dynamics associated with the initial activation position. These results highlight that, due to the increased connectivity associated with cortical folding, activation of the network is significantly slower in the networks corresponding to inflated structures. Moreover, the activation of the network is strongly affected by the site of initial activation: as shown by the width of the shaded area, for all values of activation fraction *p*, the greatest variability is observed in the intermediate inflation (middle column), while the undeformed brain displays the least variation. We attribute this to the observation that the undeformed brain—while convoluted in comparison to the inflated structures—is highly convoluted *everywhere*, whereas the intermediate brain has distinct areas of folding. That the very inflated brain displays significant variation is a consequence of the *global* geometry of the cortical surface, discussed in more detail below.

The importance of activation site on network activation is studied in more detail in Fig. 3(g–i), which shows histograms, indicating the distribution of the time taken to full-network activation (denoted t*) for each network. For all the networks studied here (see **Network activation**), full-network spreading was observed independent of topology; therefore, all simulations contributed to the results shown. These figures indicate a significant spread of activation times in all three networks: in each case, approximately a 1.5-fold difference is observed between the slowest and fastest activation time, as may be readily observed from the histograms. However, the specific features of the distribution in each case vary dramatically: for example, two clear peaks may be identified in panel (g), a single peak in panel (i), and a more complex distribution in panel (h).

The details of the activation dynamics in these networks warrants further discussion. The distributions presented in Fig. 3(g–i) arise from the competition between a global geometeric effect, and the precise details of the network obtained via the method outlined in **Network construction**. The global geometry of the cortex influences the spreading speed through its aspect ratio. Consider a uniform network defined on a prolate ellipsoid: the time to full network activation is directly related to the proximity of the initiation site to the equator, with slowest activation occurring at the poles. This effect is highly significant: as the aspect ratio of the ellipsoid increases, the activation time associated with initiation at the poles approaches a 2-fold increase, in comparison to that observed when initiated at the equator. In each case shown in Fig. 3, the cortex on which we define our network has an aspect ratio of approximately 1.5, which corresponds to the range of activation times shown in Fig. 3(g–i) described above; however, as is clear from these figures, the network structure additionally has a profound effect on the specific distribution of activation times. Figure 3(g,h) display a complex distribution associated with the locally convoluted structure of the cortex: due to the absence of such folds, Fig. 3(i) shows the expected clear peak in activation speed associated with the equator; however, due to inhomogeneities in the underlying lattice employed to construct the network (and the non-ellipsoidal shape), the distribution of activation times displays a long tail, rather than the expected smooth decay to the slowest speed associated with activation dynamics driven purely by (global) geometric effects.

To make clear the interplay between the two effects, we compared the activation in a nearest-neighbour network corresponding to the triangulation of the cortical surface, with that observed in the results above. We found that the distribution of t* in the nearest-neighbour lattice is strongly correlated with that in the cortical networks corresponding to Fig. 3 (Spearman correlation coefficient: 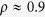 in each case; data omitted for brevity), highlighting the strong global geometric effect. However, for the undeformed brain, the correlation decreased with increasing *r*, whereas for the intermediate and inflated structures the correlation showed minimal dependence on *r* (data omitted), highlighting how the network associated with the folded nature of the cortex significantly influences the dynamics. Furthermore, the distributions of t* observed in the nearest-neighbour network and the relevant cortical network differed significantly according to the Kolmogorov—Smirnov statistic (vanishingly-small *p* value in each case).

The above results indicate that the network structure imbued by the local cortical geometry has a significant influence on the *global* network dynamics (as measured by the mean time to full network activation, t*). To characterise in more detail the link between local network dynamics and cortical geometry, we studied the network activation times associated with initiation sites centred on each node in the network, and compared these against the local Gyrification Index (lGI), which provides a measure of the cortical folding locally to each point in the network; see **Network characterisation**. We consider the time to partial network activation associated with each network node (denoted 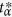, where *α* denotes the proportion of the network); such a metric enables further separation of the local network influence, and global geometrical effects (as described above). Furthermore, a more local metric such as this has the additional benefit of being of greater relevance to (say) activity propagation of the type associated with the initial stages of epileptic partial seizures, whereby spreading may initiate in a localised region, prior to spreading to wider areas of the brain (in which stage inclusion of white matter connectivity becomes of importance).

**Figure 5:**
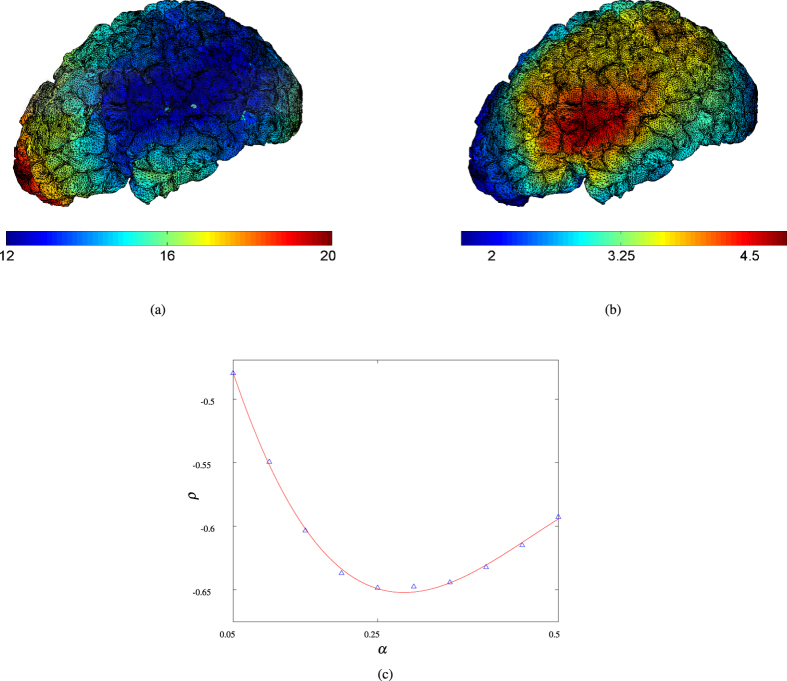
Network activation dynamics. (**a**) Heat map highlighting the relationship between initial activation site and activation speeds. Here we present 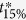, the time taken to activate 15% of network nodes from an initiation site centred on each node on the cortical surface. (**b**) Heat map displaying the local gyrification index (lGI). (**c**) The Spearman correlation between lGI and the time taken to activate a proportion *α* of the network, 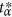, as a function of *α*. The red line here represents the best fit (in the least squares sense) of a cubic polynomial to the data.

Figure 5 shows the correlation between partial network activation and local cortical curvature. In Fig. 5(a,b) we present heat maps to highlight the initial activation sites in the cortical network which provide higher partial network activation speeds (here we consider 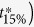, together with the corresponding lGI value. These results indicate that significant differences in (partial) network activation speed are induced by the network structure—initiation in certain regions leads to (approx.) a 2-fold reduction in activation time. Comparison of Fig. 5(a,b) indicates that increased curvature (as measured by the lGI) is linked to increases in network activation speed. We remark that we have chosen a relatively small proportion of the network, to ensure that the influence of local network structure only is captured in the activation dynamics; conversely, for reasons of clinical relevance lGI integrates a number of sulci in its measurement of cortical gyrification (see **Network characterisation**). For this reason we observe a relatively weak correlation between lGI and 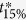 (Spearman correlation coefficient 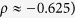. This notwithstanding, comparison between 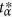 and lGI serves to further interrogate the relationship between local (network) and global effects; in Fig. 5 we present the correlation between lGI and 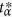, as a function of *α*. We observe the strongest correlation at *α* = 0.25, with further increases leading to minimal changes in the relationship between lGI and 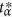. We interpret these results as follows: (i) for small *α*, the mismatch between the chosen connectivity distance resulting in network architecture associated with curvature, and that considered for lGI calculation (radius 25 mm; see **Network characterisation**) means that the correlation increases rapidly with *α*; (ii) for *α* > 0.25 global effects begin to dominate; moreover, since the distribution of high curvature is loosely aligned with the equator (of a prolate ellipsoid; see Fig. 5(b)), this correlation is similar to that induced by global effects only. We remark that the peak of 25% of the network identified in (Fig. 4) corresponds to one or two network modules; in other words, this model only using gray matter connections is appropriate for such local dynamics, but more large scale activation will be strongly influenced by the presence of inter-module white matter fibre tracts.

Lastly, we remark that the data presented in Fig. 5 correspond to a connectivity distance of *r* = 4 (*i.e.*, a relatively well-connected network inspired by physiological cortical connectivity distances); while the details differ (in particular, the link between local curvature and network structure leading to activation speed, and the cut-off at which global effects dominate), qualitatively similar results are obtained for a range of connectivity distances (details omitted for brevity).

### Cohort variability

The above detailed studies have highlighted how network connectivity associated with the highly folded structure of a specific cortical surface influences global and local network activation dynamics. To indicate the variability of such an effect within a wider population, we consider the activation of networks constructed from all brains in the NKI cohort (*n* = 121). As previously, we restrict attention to networks based on a connectivity distance of *r* = 4 and, for brevity, we consider only global activation of these networks through the time to full-network activation (t*). We remark that due to the variability in network size, the t* values obtained are normalised (arbitrarily) on the surface area of the cortex analysed in detail in **Network statistics** and **Cortical structure** so that the activation dynamics we obtain are comparable.

**Figure 6:**
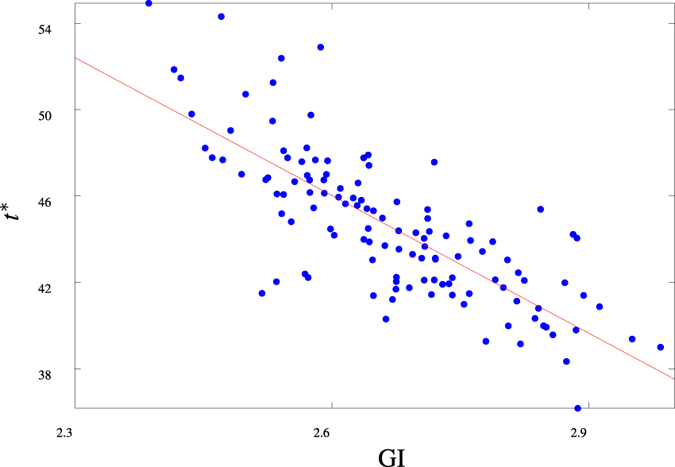
Cohort study. The mean time to full network activation t* associated with networks constructed on each brain in the NKI cohort (*n* = 121), characterised by their GI value; Spearman correlation coefficient 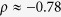.

In Fig. 6, the mean time to full-network activation for each network is presented, together with the GI value of the cortical surface on which the network was constructed. These data indicate that, in addition to the variability in network size discussed above, the cortical structure of the brains within the cohort differs significantly (with GI values lying in the range 2.4–3). Correspondingly, we observe wide variation in activation of these networks (reflecting differences in underlying structure). Importantly, this activation is strongly correlated with the degree of cortical folding, as characterised by the GI value (Spearman correlation coefficient 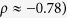. Therefore, while the details will differ, our investigations suggest that the link between cortical gyrification, network structure and activation dynamics identified previously is maintained within a larger cohort with significant differences in cortical structure.

In summary, the network analyses, and activation dynamics results that we have presented highlight the following:

1. Construction of cortical networks according to the methodology described herein (which takes account of the folded structure of the cortex by allowing for potential shortcuts through the thickness of the cortical sheet, thereby connecting cortical regions that are distant as measured on the surface) leads to significantly altered network structure, when compared to those constructed according to Euclidean distance or geodesic distance only.
2. Local differences in network structure impact significantly upon the activation dynamics of such networks. In particular, differences in partial network activation are observed, depending on the geometry of the initial activation site and its surroundings (as measured by the lGI); furthermore, global network activation is strongly correlated with the degree of cortical folding (as characterised by the GI).
3. Including communication shortcuts associated with cortical gyrification leads to networks with significantly reduced characteristic path length, and maximal node clustering (network properties associated with increased efficiencies in information/signal processing capabilities) for connection distances that coincide with those observed in histological studies of grey matter.

## Discussion

In this paper, we have investigated thoroughly the influence of the structure of a spatially embedded network on the activation dynamics of that network. While cortical folding is often studied in relation to the white matter architecture^7^, we here observe the potential funtional role of grey matter connectivity. First, the clustering coefficient is maximal and the characteristic path length minimal for organisations that represent the experimentally Known connection properties, indicating an enhancement of small-world features. Second, the folding architecture of the human brain has a significant effect on activity propagation in that some starting points on the cortical surface lead to much faster subsequent activation spreading than others. Finally, we show that the speed of information transfer is correlated with the local gyrification index of the initially activated surface region. In summary, these results indicate that connectivity along the cortical surface can influence topological and dynamic properties of human brain networks making the folding pattern an important parameter for future studies.

The networks that we study form an idealised representation of cortico—cortical connectivity; *i.e.*, we restrict attention to connectivity and activity propagation on the surface of the human cortex. The connections (and shortcuts) that we consider correspond to the dense and relatively short-range connections that exist between nearby cortical columns, and which may extend for 4–5 mm; the modelling of longer-range white matter connectivity structures—either fibre tracts connecting distant brain regions, or short association fibres (U-fibres) connecting neighbouring gyri—is not part of this work. To effect this, our method of network construction comprises a simple criterion based on Euclidean distance, modified to reject those connections which correspond to excessively long geodesic paths. In this way, tangential connections which violate biophysical ‘wiring-cost’ constraints or, equivalently, those which correspond to connections between adjacent gyri via white matter paths are prohibited.

To investigate the influence of such grey matter shortcuts on network architecture and activation dynamics, we select a cortical surface at random from the NKI dataset and construct a series of networks corresponding to an increasingly smoothed cortical structure (by an ‘inflation’ process which conserved area) and study in detail the properties of the network structure that arises from our method of network construction, in comparison to equivalent networks constructed according to Euclidean or geodesic distance only. The latter does not accommodate short cuts associated with cortical folds, while the former corresponds to a network that prohibits neither excessively long cortical paths nor white matter connectivity, and so the resulting network may not appropriately reflect physiological connectivity structures. Our results highlight that the improved connectivity associated with cortical shortcuts results in a network with significantly improved information/signal processing capabilities (as measured by the clustering coefficient and characteristic path length). In fact, we show that clustering is maximised, and characteristic path length is significantly reduced at connectivity distances that coincide with those observed in histological studies of grey matter^8^,^9^,^10^. We therefore conclude that our networks offer evidence that such a connectivity distance provides an optimal balance between communication efficiency and the associated wiring costs, and that such gains are consistent with (and enhanced by) the presence of our hypothesised short-cut connections.

The influence of the network structure discussed above on the propagation of activity through the network was highlighted by employing a simple spreading model for neural activity. The results that we presented indicate that the network structure imbued by the local cortical geometry has a significant influence on the *global* network dynamics (as measured by the time to full network activation, t*). In particular, we show that the activation of the network is strongly affected by the initiation site; furthermore, by consideration of the distribution of activation times, and comparison with those observed within a nearest-neighbour network, we highlight that the detailed network structure imbued by the cortical folds has a profound effect on global activation dynamics (while the aspect ratio of the cortex leads to an approximately 1.5-fold difference between slowest and fastest network activation in all cases, a complex distribution of network activation times is observed, associated with the locally convoluted structure of the cortex).

The link between local network structure and network dynamics is highlighted more clearly by comparison of the network activation times associated with initiation sites centred on each node in the network, and the local Gyrification Index (lGI), which provides a measure of the cortical folding locally to each point in the network. The geometric effect associated with the global aspect ratio of the brain is removed by considering the time to partial network activation, 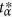 (where *α* denotes the proportion of the network) in place of the global measure t*. Our results indicate that the local curvature of the network is strongly correlated with partial network activation, and that our gray matter-based connectivity structure is appropriate for describing activation within one or two network modules (longer-range spreading being more strongly influenced by white matter connectivity not considered here). This local metric has the additional benefit of being of greater relevance to (say) activity propagation of the type associated with the initial stages of epileptic partial seizures, whereby spreading can initiate in a certain region, prior to spreading to wider areas of the brain.

In conclusion, our findings are significant for a number of reasons. Firstly, we introduce, for the first time, a network model of grey matter architecture that includes geometrically induced cortical shortcuts, the result of which is a cortical network structure that interpolates between the standard Euclidean and geodesic based structures: our method maintains much of the processing speed of the Euclidean network but restricts to more physiologically realistic structures. Secondly, by comparing elementary network characteristics such as path length and clustering, our results provide an indication of the “best” network structure in terms of balancing wiring costs and physiological constraints. In so doing, we are able to provide a simple estimate of the allowable wiring distance, which is in good agreement with available physiological data. And thirdly, our results highlight the considerable effect that initiation site has on activation dynamics within these cortical structures. In this regard, the approach we describe offers increased potential in understanding seizure disorders such as localisation-related epilepsy, in which the identification of epileptogenic brain regions is of great clinical importance. In future work, we plan to extend our model to incorporate macroscale (*i.e.* white matter) connectivity information in order to construct multiscale brain network structures that more accurately reflect neural mechanisms of relevance to seizure disorders.

## Additional Information

**How to cite this article**: Lo, Y.-P. *et al.* A geometric network model of intrinsic grey-matter connectivity of the human brain. *Sci. Rep.* **5**, 15397; doi: 10.1038/srep15397 (2015).
